# Ambient Temperature and Biomarkers of Heart Failure: A Repeated Measures Analysis

**DOI:** 10.1289/ehp.1104380

**Published:** 2012-05-15

**Authors:** Elissa H Wilker, Gloria Yeh, Gregory A Wellenius, Roger B Davis, Russell S Phillips, Murray A Mittleman

**Affiliations:** 1 Cardiovascular Epidemiology Research Unit, Beth Israel Deaconess Medical Center, Boston, Massachusetts, USA; 2 Department of Environmental Health, Harvard School of Public Health, Boston, Massachusetts, USA; 3 Division of General Medicine and Primary Care, Department of Medicine, Beth Israel Deaconess Medical Center, Boston, Massachusetts, USA; 4 Center for Environmental Health and Technology, Brown University, Providence, Rhode Island, USA; 5 Department of Epidemiology, Harvard School of Public Health, Boston, Massachusetts, USA

**Keywords:** biomarkers, climate variability, heart failure, outdoor air, susceptibility

## Abstract

Background: Extreme temperatures have been associated with hospitalization and death among individuals with heart failure, but few studies have explored the underlying mechanisms.

Objectives: We hypothesized that outdoor temperature in the Boston, Massachusetts, area (1- to 4-day moving averages) would be associated with higher levels of biomarkers of inflammation and myocyte injury in a repeated-measures study of individuals with stable heart failure.

Methods: We analyzed data from a completed clinical trial that randomized 100 patients to 12 weeks of tai chi classes or to time-matched education control. B-type natriuretic peptide (BNP), C-reactive protein (CRP), and tumor necrosis factor (TNF) were measured at baseline, 6 weeks, and 12 weeks. Endothelin-1 was measured at baseline and 12 weeks. We used fixed effects models to evaluate associations with measures of temperature that were adjusted for time-varying covariates.

Results: Higher apparent temperature was associated with higher levels of BNP beginning with 2-day moving averages and reached statistical significance for 3- and 4-day moving averages. CRP results followed a similar pattern but were delayed by 1 day. A 5°C change in 3- and 4-day moving averages of apparent temperature was associated with 11.3% [95% confidence interval (CI): 1.1, 22.5; *p* = 0.03) and 11.4% (95% CI: 1.2, 22.5; *p* = 0.03) higher BNP. A 5°C change in the 4-day moving average of apparent temperature was associated with 21.6% (95% CI: 2.5, 44.2; *p* = 0.03) higher CRP. No clear associations with TNF or endothelin-1 were observed.

Conclusions: Among patients undergoing treatment for heart failure, we observed positive associations between temperature and both BNP and CRP—predictors of heart failure prognosis and severity.

Extreme temperatures have been associated with hospitalization and death among susceptible populations (Basu et al. 2008; Bell et al. 2008; Koken et al. 2003; Zanobetti and Schwartz 2008) and individuals with congestive heart failure may be particularly vulnerable (Cui et al. 2005). Cold temperatures have been linked to hospital admissions for heart failure and incidence of cardiovascular events (Martinez-Selles et al. 2004; Stewart et al. 2002), but increases in morbidity and mortality have been observed during periods of hot weather as well (Aronow and Ahn 2004). It is possible that the biological mechanisms underlying cardiovascular health effects may vary across the range of ambient outdoor temperatures observed throughout the year. However, only a few studies have examined these associations in heart failure patients (Barclay et al. 2009; Goldberg et al. 2009). Moreover, it is not clear if short-term changes in weather patterns can be linked to preclinical changes in heart failure status.

Levels of biomarkers that reflect myocardial damage and inflammation are elevated in heart failure patients and typically rise further during episodes of acute cardiac decompensation (Fonarow 2008). B-type natriuretic peptide (BNP), a neurohormone produced principally by the ventricles of the heart in response to increasing wall stress, has been shown to be a predictor of sudden death among individuals with chronic heart failure (Berger et al. 2002) and has been associated with increased risk of readmission (Logeart et al. 2004) among individuals hospitalized for decompensation.

Elevated levels of biomarkers of systemic inflammation, immune function, and ventricular remodeling, such as C-reactive protein (CRP), tumor necrosis factor (TNF), and endothelin-1, also have been associated with morbidity and mortality among heart failure patients (Alonso-Martinez et al. 2002; Anand et al. 2005; Dunlay et al. 2008; Teerlink 2005). The evaluation of a panel of biomarkers may provide insight into underlying disease pathology and targeted therapeutic responses (Braunwald 2008). For this analysis, we investigated the association between ambient temperature and four biomarkers of heart failure in a population of individuals with stable systolic heart failure using a repeated-measures approach. We hypothesized that higher ambient and apparent temperature would be associated with elevated levels of these measures, which are associated with heart failure severity and prognosis.

## Materials and Methods

We analyzed data from a completed clinical trial that randomized 100 patients with stable heart failure and impaired systolic function to receive either 12 weeks of 1-hour group tai chi classes or time-matched heart failure education (the control) in addition to usual care. Methodological details and the primary trial results have been published elsewhere (Yeh et al. 2011). Patients who chose to participate in the study provided written informed consent; this study was approved by the institutional review boards of all participating institutions.

Participants were recruited from ambulatory clinics (primary care, general cardiology, and specialty heart failure practices) at three academic medical centers in and around Boston, Massachusetts. The inclusion criteria were physician diagnosis of chronic systolic heart failure, left ventricular ejection fraction ≤ 40% in the past 2 years; stable medical regimen that was defined as no major changes in medication in the past 3 months; and a designation of class I, II, or III for heart failure as defined by the New York Heart Association (Criteria Committee of the New York Heart Association 1994). The exclusion criteria were unstable angina or myocardial infarction in the past 3 months, major cardiac surgery within the past 3 months, history of cardiac arrest in the past 6 months, history of cardiac resynchronization therapy in the past 3 months, unstable serious ventricular arrhythmias, unstable structural valvular disease, current participation in a conventional cardiac rehabilitation program, diagnosis of peripartum cardiomyopathy within the preceding 6 months, inability to perform a bicycle stress test, lower extremity amputation or other inability to ambulate because of conditions other than heart failure, severe cognitive dysfunction (Mini-Mental State Examination score ≤ 24; Folstein et al. 1975), inability to speak English, or regular practice of tai chi.

*Biomarkers.* Blood samples were drawn from participants in a nonfasting state at the time of the study visit. BNP was analyzed in whole blood collected in EDTA (ethylenediamine-tetraacetic acid) using a commercially available point-of-service meter (fluorescence immunoassay; Biosite Triage BNP Test; Biosite Diagnostics, San Diego, CA). Serum samples were also analyzed for CRP using DPC (Diagnostic Products Corporation) Siemens Immulite high sensitivity hsCRP immunoassay (Siemens AG, Munich, Germany), for endothelin-1 using DPC Siemens chemiluminescent QuantiGLO ELISA (Siemens AG), and for TNF using Quantikine TNF-α immunoassay (R&D Systems, Inc., Minneapolis, MN). Blood samples were drawn at baseline, at 6 weeks, and at 12 weeks. The percentages of the coefficient of variation (CV%) of the intraassay and interassay for these kits were 8.8–11.6% and 9.9–12.2%, respectively, for BNP; 4.2–6.4% and 4.8–10.0%, respectively, for CRP; 3.1% and 6.7%, respectively, for endothelin-1; and 5.3% and 8.4%, respectively, for TNF.

*Pollution and weather data.* Ambient temperature, relative humidity, dew point temperature, and barometric pressure were obtained from the National Weather Service (National Climatic Data Center 2011) daily summaries of meteorological data measured at Logan International Airport (Boston, MA). Apparent temperature is a metric used to describe how people perceive the combination of temperature and humidity (Steadman 1984). The values for apparent temperature are based on the measures of ambient and dew point temperature and were calculated using the following formula:

Apparent temperature = –2.653 + [0.994 × 24-hr mean air temperature (°C)] + [0.0153 × 24-hr mean dew point temperature (°C)^2^] [1]

We obtained hourly measures of ambient fine particulate matter (PM ≤ 2.5 µm in aerodynamic diameter; PM_2.5_) from the Harvard Countway Library SuperSite (Boston, MA) and ozone from the averages of five U.S. Environmental Protection Agency monitors located within the Boston Area.

*Statistical methods.* The biomarker data (CRP, BNP, endothelin-1, and TNF) were examined using summary statistics and distributional plots to identify outliers and assess normality. Log-transformation of biomarkers was performed for all analyses. Outliers were defined as values falling outside 1.5 times the interquartile range of the data. There were two outliers identified for endothelin-1 and one outlier for TNF. Analyses were conducted including all observations and also excluding outlier observations.

Separate models were constructed for one to four day moving averages of ambient and apparent temperature for each biomarker using fixed-effects regression in R (version 2.9; R Foundation for Statistical Computing, Vienna, Austria) with the *plm* package for repeated-measures data. Unlike mixed-effects models commonly used in unbalanced repeated-measures analyses, which borrow information at the population level to account for within- and between-person variability, fixed-effects regression controls for all between-person differences by estimating subject-specific intercepts, thus isolating the within-person estimates of association. Although this approach is more commonly used in the field of econometrics (Hausman 1978; Hausman and Taylor 1981), it also has previously been used in air pollution studies (Gold et al. 2000; Rückerl et al. 2006). In this model, each participant forms their own stratum analogous to a conditional logistic regression framework for binary outcomes in a case-crossover study. The model provides estimates for the time-varying covariates only, because time invariant covariates do not change within person. The model takes the general form

*y_it_* = *X_it_*β + α*_i_* + *e_it_*, [2]

where *y_it_* is the outcome *y* at time *t* for the *i*th subject, *X_it_* is a matrix of time-varying predictors, α*_i_* is a random intercept for each subject, and *e_it_* is the error term. In the primary analyses, ambient and apparent temperature were modeled as continuous linear functions. All models were adjusted for time and seasonality using a harmonic function [sine (2π × day of year/365.25) and cosine (2π × day of year/365.25)], day of week, and body weight at each visit as a marker of hydration status. Models of ambient temperature were additionally adjusted for corresponding moving-average values of relative humidity and barometric pressure. Models of apparent temperature were adjusted for barometric pressure only because the formula for apparent temperature accounts for the dew point temperature. We also tested for effect modification by diabetes status, sex, and randomization to the tai chi treatment arm of the study using cross-product terms and examining statistical significance at *p* < 0.05.

In a sensitivity analysis, we adjusted for corresponding moving averages of PM_2.5_ and ozone in separate models. We also included both linear and quadratic terms for relative humidity and pressure. To further explore potential nonlinear relationships, we used the *mgcv* package for generalized additive models (version 1.6; R Foundation for Statistical Computing) to fit penalized splines. We hypothesized that changes in temperature could be associated with different physiologic responses in summer and winter. Therefore, we performed sensitivity analyses by subsetting to season when visits began, defined as warm (March through August) or cool (September through February) seasons and also tested for interactions using a cross-product term and examining statistical significance of this term at *p* < 0.05. In a sensitivity analysis, we also fit linear-mixed models with random effects for all four biomarkers using the *plm* package. No material difference was observed, so we present results for fixed effects only. All results are presented as a percent change for a 5°C increase in either ambient or apparent temperature measure with 95% confidence interval (CI). This increment was chosen to be representative of day-to-day variability in temperature in the Boston area.

## Results

**Figure 1:**
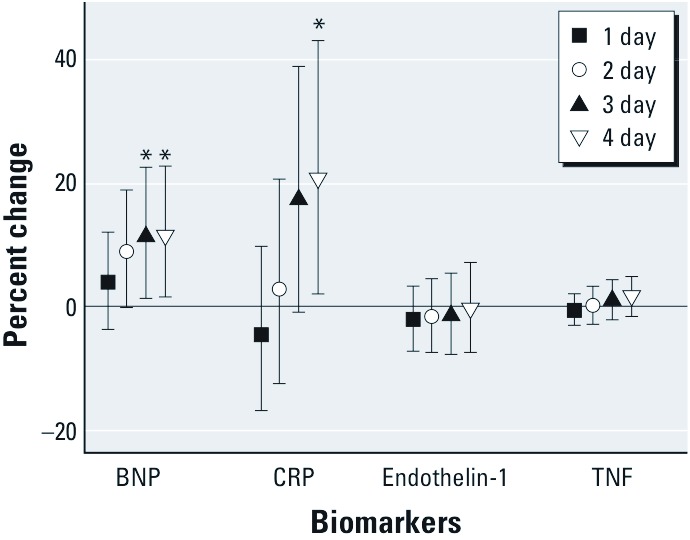
The percent change and 95% CIs for BNP, CRP, endothelin-1, and TNF for 1- to 4-day moving averages. Estimated changes in biomarker levels associated with a 5°C change in moving average temperature over the previous 1–4 days. Plots indicate point estimate and 95% CIs. All models are adjusted for day of week, sine, and cosine of day of year, body weight, and atmospheric pressure. BNP models include 100 participants with 285 measures; CRP models include 81 participants with 230 measures; endothelin-1 models include 93 participants with 186 measures (two outlier observations were not included); and TNF models include 99 participants with 269 observations (one outlier observation was not included). *p < 0.05.

In Table 1, we show the characteristics of the study population. Participants were predominantly male (64%) and white (86%). The age at baseline ranged from 34 to 96 years (mean ± SD of 67.4 ± 12). Table 2 summarizes the daily meteorological data during the study period. Ambient temperature ranged from –11 to 27°C throughout the study period (mean ± SD of 12.5 ± 9.0) and was highly correlated with apparent temperature (Spearman correlation > 0.9). The median of the within-person range in ambient temperature across study visits was 13°C. The largest within-person range in both ambient and apparent temperature was observed for participants who began the study in mid-September, when Boston weather patterns may still be quite like summer, and who completed the study in mid-December. Distributions of the biomarkers and their correlations are described in Table 3 by visit. All 100 participants in the study provided baseline blood samples for the BNP assessment. Three samples were available for 89 of these participants. There were four participants who provided only one sample. Endothelin-1 measures typically occurred at visits 1 and 3, but 8 samples were analyzed at visit 2 when visit 1 or visit 3 data were unavailable. Figure 1 shows the results from the fully adjusted models of apparent temperature. Higher apparent temperature was associated with higher levels of BNP beginning with 2-day moving averages and reached statistical significance for 3- and 4-day moving averages. Specifically, we observed that a 5°C change in 3- and 4-day moving averages of apparent temperature were positively associated with an 11.3% higher (95% CI: 1.1, 22.5; *p* = 0.03) and an 11.4% higher (95% CI: 1.2, 22.5; *p* = 0.03) BNP, respectively. CRP results followed a similar pattern, but were delayed by 1 day; CRP levels were significantly higher after 4-day moving averages, and a 5°C change in apparent temperature was associated with 21.6% higher CRP (95% CI: 2.5, 44.2; *p* = 0.03). No clear association was observed between apparent temperature and endothelin-1 or TNF. Inclusion of outliers did not materially change these findings. Similar results were observed for ambient temperature for all outcomes (data not shown). We found no evidence of effect modification by season, diabetes status, sex, or randomization to tai chi treatment using cross-product terms and a *p*-value of 0.05 as the criterion for significance (data not shown).

**Table 1:** Patient characteristics^a^ at initial visit.

Characteristic	Mean ± SD or no. of participants
Age (years)	67 ± 12.0
Sex (female)	36
Ethnicity/race	
Nonwhite	14
Income > $50,000	42
Refused to answer	14
Smokingb	10
Alcohol useb	48
Left ventricular ejection	
fraction	29.0 ± 7.6
NYHA class heart failure	
I	20
II	63
III	17
Cardiovascular comorbidities	
Myocardial infarction	58
Diabetes mellitus	35
Hypertension	70
Previous procedures	
Coronary artery bypass graft	36
Valve repair/replacement	14
Stent	49
Medications	
Beta blockers	86
Ace-inhibitors/angiotensin receptor blockers	85
an = 100 participants. bCurrent use (yes/no).	

**Table 2:** Pollution and meteorology for study visit days (n = 285).

Pollution and weather	Mean	SD	Median	Minimum	Maximum
Ambient temperature (°C)		13		9		14		–11		27
Apparent temperature (°C)		12		10		13		–7		30
Relative humidity (%)		66		15		66		30		99
Barometric pressure (mmHg)		761		7		760		740		776
Ozone (ppb)		24		10		22		30		66
PM2.5 (µg/m3)		8.7		5.3		7.2		0.2		29.9

**Table 3:** Description of inflammatory markers at each visit.

Visit 1					Visit 2					Visit 3				
Biomarkers	n	Geometric mean	Median (25th, 75th percentile)	n	Geometric mean	Median (25th, 75th percentile)	n	Geometric mean	Median (25th, 75th percentile)
BNP (pg/mL)		100		112.48		103.5 (44.3, 284.5)		92		97.71		90.4 (40.9, 238)		93		106.54		110 (46.7, 223)
CRP (mg/L)		81		2.81		3.1 (0.94, 6.89)		76		2.17		1.84 (0.92, 5.60)		73		2.58		1.88 (0.93, 7.86)
Endothelin-1 (pg/mL)a		90		2.31		2.32 (1.80, 2.90)		8b		1.813		1.77 (1.56, 2.03)		90		2.42		2.39 (1.94, 2.92)
TNF (pg/mL)a		95		1.70		1.65 (1.11, 2.07)		89		1.65		1.59 (1.14, 2.19)		86		1.62		1.47 (1.19, 2.02)
aOutlier observations are included. bEndothelin-1 measures typically occurred at visits 1 and 3, but 8 samples were analyzed at visit 2 when visit 1 or visit 3 data were unavailable.																		

In the analyses examining the exposure–​response relationships between temperature and biomarkers, we did not observe substantial deviations from linearity [see Supplemental Material, Figure S1 (http://dx.doi.org/10.1289/ehp.1104380)]. We observed no clear evidence of nonlinear relationships, and none of the spline terms were statistically significant (all *p* > 0.05). Adjustment for PM_2.5_ or ozone as potential confounders did not substantially alter our results (data not shown).

## Discussion

In this analysis of the association between temperature measures and biomarkers related to inflammation and cardiovascular function in a population of heart failure patients, we observed higher levels of BNP beginning with 2-day moving averages. These results reached statistical significance for moving averages of 3 and 4 days. CRP results followed a similar pattern but were delayed by 1 day. These two biomarkers reflect interrelated mechanisms known to be associated with heart failure prognosis and symptom severity. CRP is a an indicator of systemic inflammation and is associated with risk of heart failure decompensation (Sato et al. 1999), whereas BNP is more specifically a marker of increasing hemodynamic load used in diagnosis and risk stratification among patients with congestive heart failure (Koglin et al. 2001). Predischarge BNP is a strong independent predictor of postdischarge outcome (Logeart et al. 2004) and long-term prognosis (Latini et al. 2002). To our knowledge, this is the first evidence that within-person BNP levels may be elevated during episodes of increased temperature. The results reported in this study are scaled to a 5°C change. Because we were examining associations across a season, the within-person differences in temperature could be quite large. We selected a 5°C change to represent the typical day-to-day variability in temperature within the Boston area. This is well within the range of data that we observe and represents a relatively small within-person change.

The levels of the inflammatory markers we observed in our study population of individuals with heart failure were elevated, as expected in a population of heart failure patients. For example, a BNP level of 100 pg/mL is typically used as a clinical cutoff for diagnosis of heart failure and decompensation, and the prognostic value of elevated plasma BNP in symptomatic systolic heart failure has been consistently reliable in various clinical settings (Cheng et al. 2001). For context, the magnitude of the association between temperature and BNP observed in this study indicates that 10°C higher temperature is associated with approximately one-third of the impact of a transient increase in sodium load from 1,610 mg/day to 5,750 mg/day for subjects with established heart failure, a stimulus that may trigger decompensation in some patients (Damgaard et al. 2007).

Most prior studies of environmental determinants in heart failure patients have focused on hospitalizations and mortality rather than symptoms and preclinical changes. Studies that have examined seasonal trends in hospital admissions and mortality for heart failure have typically observed a winter peak (Barnett et al. 2008; Boulay et al. 1999; Stewart et al. 2002). In a recent study, Gotsman et al. (2010) examined the association between temperature and prognosis in heart failure patients. They showed greater rates of hospitalization in the winter time but also found that warmer weather and admission during summer months were positively associated with symptom severity, prognosis, and reduced survival. To our knowledge, there is only one panel study that has directly examined health effects of temperature in heart failure patients. In this study, which was conducted in Montreal, Quebec, Goldberg et al. (2008) found an inverse association between oxygen saturation and maximum air temperature and relative humidity. They also found that maximum ambient air temperature, higher relative humidity, and ozone on the concurrent day predicted poorer self-reported general health (Goldberg et al. 2009). In our analyses of associations between temperature and biomarkers of heart failure, we observed positive associations with higher air temperature moving averages but not with relative humidity or ozone. This result may be due to the differences in the outcomes and also the differences in time windows assessed, Goldberg only tested lags of 0–2 days (3-day average). Our findings suggest an association with BNP after only two days, but it is possible that longer integrated averages of temperature over several days, which reflect a period of prolonged elevated temperature, are necessary to observe upregulation of CRP.

We are not aware of any previous studies investigating the effects of temperature with BNP or with CRP in heart failure patients. However, prior studies have reported the associations between temperature and CRP in other populations, and the results have been mixed (Halonen et al. 2010; Hampel et al. 2010; Schneider et al. 2008). Halonen et al. (2010) estimated temperature effects in a relatively healthy cohort of men and also observed inverse associations between temperature and CRP for lags of 0–1 day and for moving averages of up to 4 weeks. Schneider et al. (2008) measured CRP within a population of myocardial infarction survivors and observed inverse associations with temperature for averages and lags < 1 week. However, a more recent study by Hampel et al. (2010) contrasted patients with coronary heart disease and pulmonary disease in their responses to temperature and found no association with CRP among patients with pulmonary disease and lower CRP associated with lower temperature (corresponding to a positive association) for almost all lags among coronary heart disease patients. As has been suggested by these authors, differences in populations and their comorbidities and patterns of medication use may explain discrepant findings (Hampel et al. 2010).

We did not observe any evidence of associations with either TNF or endothelin-1 and temperature. Endothelin-1 is released from endothelial cells and has strong vasoconstrictive properties. Higher levels of endothelin-1 have been associated with heart failure severity and higher mortality rates (Bolger et al. 2002; Wei et al. 1994; Yang et al. 2005). In this analysis of only two measures per subject, we may not have had sufficient power to detect within-person changes. We note that 90% of endothelin-1 is synthesized in the endothelium and acts on the vascular smooth muscle locally (Levin 1995). TNF is a cytokine associated with inflammatory properties that also has been associated with severity and risk stratification of heart failure in a number of studies (Levine et al. 1990; Sharma et al. 2000). Our results are consistent with those of Halonen et al. (2010) who did not observe any significant association between temperature and TNF among older men also residing within the same region as our study participants.

Although we addressed the shape of the exposure–response relationship using penalized splines in additional analyses, we did not observe any statistically significant deviation from linearity. The only models with estimated degrees of freedom (df) > 1 were for endothelin-1, and these associations were nonsignificant (the estimated 1-day moving average df was 2.3; *p* = 0.4). In our analyses, neither PM_2.5_ nor ozone confounded the association between temperature and biomarkers and were not significantly associated with biomarker levels. We previously observed no consistent association between BNP and PM_2.5_ in a similar population of heart failure patients in the Boston area (Wellenius et al. 2007). Similar results were also observed in Aberdeen, Scotland, where there was no change in the number of hematological parameters measured in response to air pollution exposures in a panel of 132 heart failure patients (Barclay et al. 2009). However, our findings differ from other studies that have shown that PM_2.5_ is associated with changes in inflammatory biomarker levels in older aging populations (Dubowsky et al. 2006; Wilker et al. 2011), individuals with diabetes (O’Neill et al. 2007), and persons with coronary artery disease (Delfino et al. 2008). Heart failure patients may spend less time outside and have different time-activity and exposure patterns from healthy individuals and from those with other chronic conditions (Oka et al. 1993; van den Berg-Emons et al. 2001). These differences could also potentially explain our findings of no substantial deviations from linearity in the exposure–response relationship, if these patients with chronic heart failure are more vulnerable to extreme heat and spend little time outdoors during periods of extreme cold.

Potential limitations of this study include a limited number of repeated measures on study participants. This study specifically enrolled patients with heart failure and systolic dysfunction. Therefore, the results may not be generalizable to patients with heart failure and preserved systolic function. Additionally, the purpose of the original study was to examine effects of tai chi. This intervention was found to be associated with improved quality-of-life measures, but randomization to tai chi was not associated with statistically significant changes in the levels of these biomarkers (Yeh et al. 2011), and we did not observe any evidence of effect modification by tai chi randomization status in our analysis. We are also limited in our ability to assess spatial heterogeneity of the temperature metric, because we used only one stationary site for our analyses. Most (88%) participants in our study population lived within 40 km of Boston and our central site monitor; therefore, we expected to have limited misclassification for our within-person analyses. However, this study did not collect information on air-conditioner usage and availability, which also may be a source of variability in heat-related physiologic responses (Curriero et al. 2002; O’Neill et al. 2005; Ostro et al. 2010).

As mentioned above, time-activity patterns (Oka et al. 1993; van den Berg-Emons et al. 2001) could potentially confound or modify associations between temperature and these biomarkers, but we did not have sufficient data to address this topic. It is also possible that hemoconcentration could, in part, explain the associations between temperature and biomarkers, in situations where participants become more dehydrated in warmer weather. Data on blood urea nitrogen or hematocrit were not available in this study, but we did include body weight at each visit as a marker of hydration status in heart failure patients and found that the changes in the estimated association between temperature and the biomarkers were negligible (< 5%).

In this study of 100 patients with heart failure and systolic dysfunction, we observed significantly higher BNP and CRP, which are biomarkers related to heart failure symptoms and prognosis, with 3- to 4-day moving averages of apparent temperature. These findings suggest that changes in temperature and meteorology may alter underlying physiologic responses in this vulnerable population. Because of their compromised hemodynamic response, individuals with heart failure may have increased stress in the ventricles, as reflected by a rapid increase in circulating BNP levels, which may be accompanied or perhaps followed by elevation in systemic inflammatory responses, as indicated by CRP. Given the large public health burden of heart failure, further work is needed to examine the role of ambient temperature in influencing preclinical changes that occur before an episode of acute heart failure decompensation.

## Supplemental Material

(168 KB) PDF
