# Supplementary material for: Ambient Temperature and Biomarkers of Heart Failure: A Repeated Measures Analysis
Source: Environ Health Perspect. 2012 May 15;120(8):1083–7. doi: 10.1289/ehp.1104380 (PMC3440076; doi:10.1289/ehp.1104380)
Supplement: (168 KB) PDF [file ehp.1104380.s001.pdf]

Supplemental Material

Ambient Temperature and Biomarkers of Heart Failure: A Repeated Measures Analysis

Elissa H. Wilker[1,2], Gloria Yeh[3], Gregory A. Wellenius[4], Roger B. Davis[3], Russell S. Phillips[3]
Murray A. Mittleman[1,2]

Supplemental Material, Figure S1

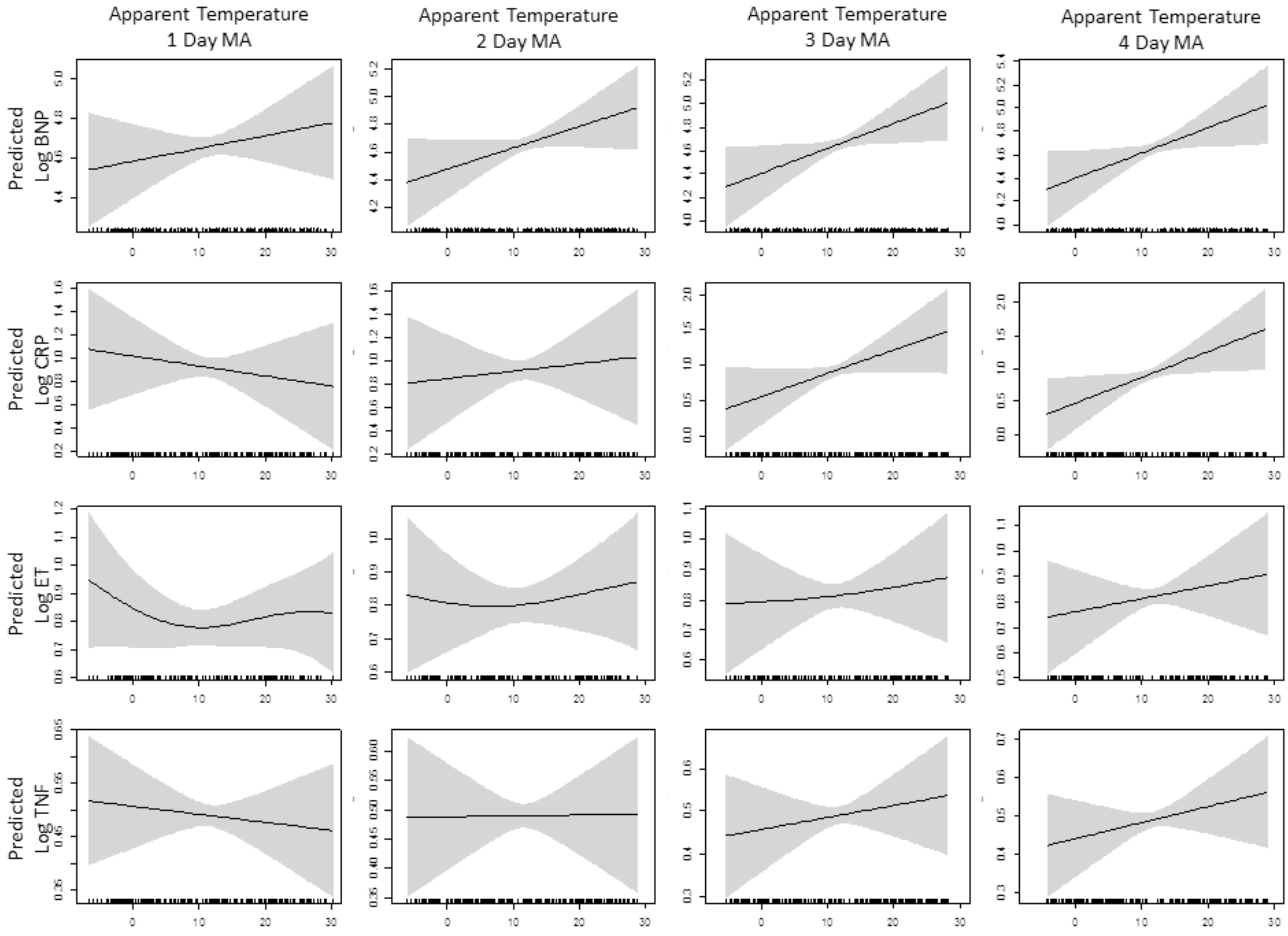

Legend

Penalized spline representations of the association between each study biomarker and moving averages (MA) of apparent temperature. In each plot the solid line denotes the fitted spline term for the mean predicted value of the biomarker as a function of apparent temperature (x-axis, in °C). The shaded areas denote the 95% confidence intervals for the smoothed term. These plots indicate that there was no evidence of statistically significant departures from linearity for the association between any of the study biomarkers and apparent temperature. ET= Endothelin-1; TNF=Tumor Necrosis Factor.
